# Telemedicine Solutions for Chronic Pain: Design, Implementation, and Evaluation of a Novel System

**DOI:** 10.5812/aapm-165256

**Published:** 2025-09-30

**Authors:** Shahabedin Rahmatizadeh, Zeinab Kohzadi, Ali Dabbagh, Hassan Emami, Zahra Kohzadi, Mehrdad Taheri

**Affiliations:** 1 Department of Health Information Management and Technology, School of Allied Medical Sciences, Shahid Beheshti University of Medical Sciences, Tehran, Iran; 2 Department of Anesthesiology, Anesthesiology Research Center, Shahid Modarres Hospital, School of Medicine, Shahid Beheshti University of Medical Sciences, Tehran, Iran; 3 Ilam County Health Center, Ilam University of Medical Sciences, Ilam, Iran; 4 Department of Anesthesiology, Imam Hossein Hospital, School of Medicine, Shahid Beheshti University of Medical Sciences, Tehran, Iran

**Keywords:** Telemedicine, Chronic Pain, Pain Management, Internet-based Intervention, User-Centered Design

## Abstract

**Background:**

Chronic pain is a complex condition affecting a significant portion of the population, and various approaches are being explored for its management.

**Objectives:**

This study aims to design and evaluate a telemedicine system for chronic pain management.

**Methods:**

A developmental cross-sectional study was conducted in three phases: Identifying a minimum dataset through literature review and expert opinion, developing and testing a prototype using Nielsen's Ten Heuristics, and finalizing and evaluating the system with the Questionnaire for User Interface Satisfaction (QUIS).

**Results:**

The minimum dataset included 56 elements across eight categories. The system was developed as a web-based platform. Usability evaluation based on Nielsen’s ten principles showed that the highest number of issues (n = 9) and the greatest severity (mean score 2.37) were related to the system-real world consistency. User satisfaction with the interface was favorable, with mean scores of 8.07 ± 0.41 for specialists and 7.73 ± 0.55 for patients.

**Conclusions:**

The designed telemedicine system for chronic pain management, considering its features, provides specialized services to patients with chronic pain who are unable to visit a pain specialist in person. Additionally, by eliminating unnecessary visits to medical centers, this system can be beneficial in reducing related costs.

## 1. Background

According to the definition of the International Association of Pain (IASP), pain is an unpleasant sensory and psychological experience that is related to potential or actual tissue damage (1), and chronic pain is persistent or recurrent pain that lasts for more than 3 months (2). Chronic pain is highly complex and involves symptoms and complications such as activity avoidance, job loss, decreased performance, reduced quality of life, decreased mobility, impairment in physical health, and the psychological and economic well-being of patients, as well as sleep disorders (3). The prevalence of chronic pain in the general population typically ranges from 13% to 55%. Most studies report prevalence rates between 13% and 25% (4, 5), while the Centers for Disease Control and Prevention (CDC) has estimated its prevalence at 20.4% (6). Chronic pain imposes substantial costs on both individuals and society, with chronic pain patients utilizing primary healthcare services five times more frequently than the general population (7, 8).

Management of chronic pain involves continuous treatment and control that extends over a long duration (9). It often requires a multidisciplinary approach, which may include pharmacotherapy, physiotherapy, psychological interventions, lifestyle modifications, and complementary therapies (9, 10). The primary goal of chronic pain management is to enhance the quality of life for individuals suffering from chronic pain by reducing pain levels, improving physical function, and addressing the emotional consequences of pain (9, 11). Most of the patients who suffer from chronic pain have mobility limitations (12, 13). One of the challenges of managing chronic pain is a lack of access to specialized care (14). Information and communication technologies (ICTs) offer a promising solution to improve access to affordable, high-quality health care (15). Telemedicine, by utilizing ICTs, can overcome geographical barriers, which is particularly valuable for rural and underserved populations in developing countries (15, 16). Telemedicine services have demonstrated significant potential in providing high-quality, patient-centered care for chronic pain management (17).

Studies have been conducted in this field. In the study by Dharmalingam et al., the perceptions of patients with chronic pain who received remote treatment were evaluated, along with their self-efficacy and level of pain coping. More than half of the patients reported that telemedicine was beneficial for managing their pain (18). In the study by Tumturk et al., the home-based remote rehabilitation program was found to be superior to the home group paper-based program in terms of pain, function, quality of life, and proprioception in patients with knee osteoarthritis (19). Yarns et al. evaluated telehealth-delivered video-based emotional awareness and expression therapy (VEAET) for older veterans with chronic musculoskeletal pain, finding significant pain reduction from baseline to follow-up (20). Nowadays, many countries are implementing comprehensive and extensive telehealth programs within their healthcare systems (21). In developing countries, due to financial constraints, lack of expertise, shortage of doctors, and inadequate roads and transportation facilities, telemedicine can be a suitable solution to address these issues, especially in underserved and remote areas (22). Although Iran’s primary healthcare system has improved, several major differences remain because of issues related to region, economy, and organization. To solve these problems, we should have targeted laws, increase resource use, and develop innovative ideas to make quality healthcare available to all, regardless of where they live (23-25).

## 2. Objectives

Telemedicine has proven beneficial and can address the healthcare needs of underserved areas in Iran. Since no system has existed for this purpose in Iran so far, this study aims to design and evaluate a telemedicine system for chronic pain management.

## 3. Methods

This was a developmental cross-sectional study conducted from May to December 2024 at Shahid Beheshti University of Medical Sciences, Tehran, Iran. The study was carried out in three main phases: (1) Identification of the minimum dataset, (2) design and implementation of the system, and (3) evaluation of usability and user satisfaction.

In the first phase, articles were initially searched in many trusted databases to compile a list of possible data elements for chronic pain management (26-29). An expert panel of eight pain physicians, anesthesiologists, and general practitioners then participated in a two-round Delphi study to review and refine the list. Researchers selected experts through purposive sampling, ensuring all had worked as clinicians for at least five years, had knowledge of managing chronic pain, and were willing to participate. Two weeks were allowed between each round of Delphi voting.

The questionnaire was designed using a 5-point Likert scale ranging from strongly agree (score 5) to strongly disagree (score 1) to assess the perceived necessity of data elements. The questionnaire comprised eight categories: Demographic information, patient history, pain history, a list of psychological elements and activities of daily living, diagnosis and treatment, paraclinical tests, laboratory tests, and examinations. An open-ended question was included at the end of the questionnaire for each section of data elements to allow experts to suggest additional data elements if they deemed necessary. The questionnaire was distributed to experts in the field in both paper and online formats.

Once expert feedback on the perceived necessity of the datasets was collected, the data was analyzed using descriptive statistics and SPSS software version 18. Based on the results of the questionnaire analysis, non-essential data elements that did not receive a satisfactory score were removed from the minimum dataset. Finally, the information elements that were deemed essential for recording information related to chronic pain management and that should be included in the system's database were identified. The decision to include or exclude data elements was based on the scores calculated from expert feedback. If the consensus among experts was less than 50%, the data item was removed. If the score was higher than 75%, the data item was considered approved. If a question received a score between 50 and 75%, it proceeded to a second Delphi round for further evaluation.

In the second phase, following the analysis of the data obtained from the Needs Assessment Questionnaire, the initial version of the system was developed in the visual studio code environment using NodeJS/JavaScript and Python programming languages, along with the MySQL database. To evaluate the usability and identify potential design and user interface problems in the initial prototype of the system, a heuristic evaluation was conducted using Nielsen's ten usability heuristics: Visibility of system status, match between system and the real world, user control and freedom, consistency and standards, error prevention, recognition rather than recall, flexibility and efficiency of use, aesthetic and minimalist design, help users recognize, diagnose, and recover from errors, and help and documentation.

Three medical informatics experts, each with over five years of experience and knowledge of human-computer interaction, were selected using a convenient and targeted approach to evaluate the system through a usability test. The evaluators independently assessed different parts of the system for adherence to Nielsen's ten heuristics. They identified and documented any problems in a data collection form. This form included the problem name, a complete description of the problem, the location of the problem, the violated usability principle, and the severity of the problem. The evaluators thoroughly discussed and debated the identified usability problems and their alignment with each of Nielsen's ten heuristics. Any disagreements were resolved through consensus.

The final list of problems was provided to the evaluators, who independently assessed the severity of each problem. The severity of the problem was determined based on three criteria: Repetition, continuity, and impact. For the ranking of the found problems, one of the degrees of not a problem at all (score 0), cosmetic problem only (score 1), minor problem (score 2), major problem (score 3), and catastrophic problem (score 4) was assigned (30). The actual severity of each problem was determined by the average score of the evaluators. Finally, the identified problems were categorized based on the average severity obtained into one of five categories: Not a problem at all (score 0 - 0.5), cosmetic problem only (score 0.6 - 1.5), minor problem (score 1.5 - 2.5), major problem (score 2.6 - 3.5), and catastrophic problem (score 3.6 - 5).

In the third phase, the AramDard system was developed. Users accessed the system through dedicated panels designed for patients, medical staff, and administrators. The system enabled members to use electronic health records, submit consultation requests, upload documents, schedule appointments, follow up on referrals, and send direct messages to their doctors. Structured forms were also provided to allow the submission of paraclinical images and PDF files. The Questionnaire for User Interface Satisfaction (QUIS) was employed to evaluate user satisfaction with the system's interface (31).

This questionnaire had 30 questions; 3 questions related to the participant's identity information and 27 other questions related to the evaluation of usability and satisfaction in 5 parts: Comments related to overall reactions to the software (6 questions), screen (4 questions), terminology and system information (6 questions), learning (6 questions), and system capabilities (5 questions) (31). Each question had an answer with a score of 0 to 9, where the number 0 indicates the lowest level of ability and satisfaction, and the number 9 indicates the highest level of ability and satisfaction (a score of 0 - 3 is weak, 3 - 6 is moderate, 6 - 9 is good). Each user completed this questionnaire online and provided it to the researcher.

**Figure 1.**
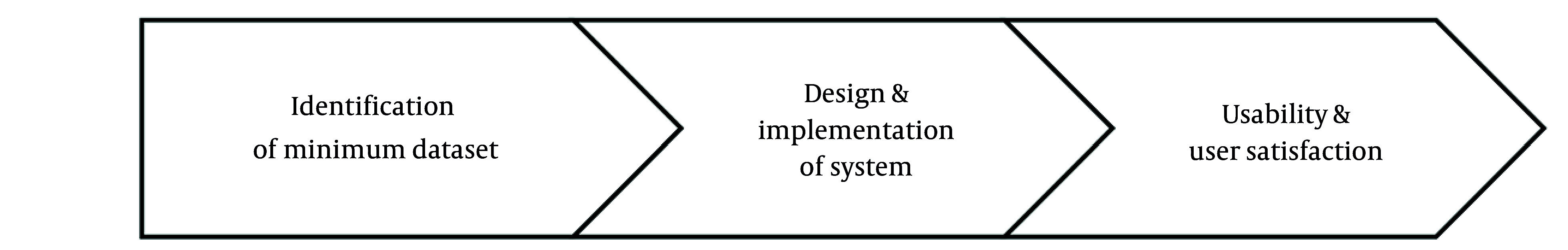
Summary of the implementation method

In this study, 15 patients with chronic pain and 7 pain management specialists completed the QUIS Questionnaire. Patient inclusion criteria were age ≥ 18 years, a diagnosis of chronic pain, the ability and expected use of the telemedicine platform, and willingness to participate, while patients with severe cognitive impairment or inability to provide informed consent were excluded. Specialists were selected based on relevant experience in pain management (at least 5 years) and their willingness to participate in the system evaluation and complete the QUIS Questionnaire. The summary of the implementation method is shown in Figure 1. 

This research was approved by the Ethics Committee of Shahid Beheshti University of Medical Sciences (ethics code: IR.SBMU.RETECH.REC.1403.026). According to the approval granted by the Ethics Committee, verbal informed consent was obtained from all participants prior to their inclusion in the study. For each participant, verbal consent was documented at the time of the interview by the principal researcher, following procedures approved by the Ethics Committee, using an internal data collection form; This form included participant codes, interview dates, and confirmation of consent. Additionally, a member of the research team was present to witness the consent process. Since all data were collected and stored anonymously, written consent was deemed unnecessary. Prior to participation, participants were fully informed about the study’s objectives, procedures, and their right to withdraw at any time. Participants explicitly communicated their agreement to the research team.

### 3.1. Data Analysis

Data analysis was conducted using SPSS version 18. Descriptive statistics, including means, standard deviations, and frequency distributions, were calculated to summarize participant characteristics and responses to the QUIS Questionnaire. User satisfaction was assessed based on average scores and response distributions. Usability problems identified during the heuristic evaluation were categorized according to the heuristics they violated, and both their frequency and mean severity were computed.

## 4. Results

The specialist physicians involved in the phase of determining the minimum data set consisted of 8 individuals. The number of females (62.5%) was greater than that of males. The most common specialization was in pain (50%). The most common range of work experience was between 10 and 20 years (37.5%). Their demographic characteristics are summarized in Table 1. 

**Table 1.** Demographical Characteristics of Participants (N = 8)

Variables	No. (%)
**Gender**	
Male	3 (37.5)
Female	5 (62.5)
**Type of specialization**	
General practitioner	1 (12.5)
Pain specialist	4 (50)
Anesthesiologist	3 (37.5)
**Work experience (y)**	
Less than 5	2 (25)
Between 5 and 10	1 (12.5)
Between 10 and 20	3 (37.5)
More than 20	2 (25)

After reviewing the literature and sources, 55 data elements were provided to the experts. In the first round of the Delphi study, childbirth history scored between 50 and 75. Additionally, in the open-ended questions section of the questionnaire, the experts added two elements: City of residence and occupation. These two additional data elements, along with childbirth history, were provided to the experts in the second round of the Delphi study. Childbirth history was scored less than 75 and was subsequently removed. Therefore, the minimum data set was determined to consist of 56 elements categorized into 8 groups: Demographic information, patient history, history of pain, paraclinical tests, psychological and activities of daily living, diagnosis and treatment, and laboratory tests. Table 2 shows the minimum data set obtained in the first and second rounds of the Delphi study.

**Table 2.** List of Minimum Data Set

Data Element	Strongly Agree	Agree	Undecided	Disagree	Strongly Disagree
**Demographic information**					
First name and last name	8	-	-	-	-
Age	7	1	-	-	-
Gender	7	1	-	-	-
Education	6	-	2	-	-
Height	6	1	1	-	-
Weight	7	1	-	-	-
Occupation	7	1	-	-	-
National code	8	-	-	-	-
Medical file number	8	-	-	-	-
Phone number	8	-	-	-	-
Type of insurance	6	2	-	-	-
City of residence	6	1	1	-	-
E-mail	6	2	-	-	-
Address	8	-	-	-	-
**Patient history**					
Cancer in the individual	6	1	1	-	-
Cancer in the family	6	1	1	-	-
Injection drug use	6	1	1	-	-
Direct intraspinal injections	7	1	-	-	-
Smoking	6	2	-	-	-
Local anesthesia	7	1	-	-	-
Other diseases	6	1	1	-	-
Drug allergy	6	1	1	-	-
**History of pain**					
Location	8	-	-	-	-
Pain duration	7	1	-	-	-
Onset of pain	6	2	-	-	-
Pain intensity	8	-	-	-	-
Kind of pain	7	1	-	-	-
Quality of pain	6	2	-	-	-
Suspected etiology	6	1	1	-	-
The spread of pain to other organs	8	-	-	-	-
Intensity of pain during movement	7	1	-	-	-
Frequency in the past 6 months	7	1	-	-	-
Pain relievers and exacerbating factors	7	1	-	-	-
**Paraclinical tests**					
CT	8	-	-	-	-
MRI	8	-	-	-	-
SPECT	8	-	-	-	-
Radiology	8	-	-	-	-
EMG	8	-	-	-	-
BMD	8	-	-	-	-
**Laboratory tests**					
CBC	6	1	1	-	-
CRP	7	1	-	-	-
ESR	7	1	-	-	-
LDL	6	1	1	-	-
TSH	6	2	-	-	-
D3	8	-	-	-	-
Calcium	8	-	-	-	-
Magnesium	8	-	-	-	-
**Psychological and activities of daily living**					
Pain interference on a daily activity	7	1	-	-	-
Impact of pain on occupational tasks	6	2	-	-	-
Impact of pain on sleep	6	1	1	-	-
Impact of pain on mood, anxiety, and depression	7	1	-	-	-
**Diagnosis and treatment**					
Diagnosis	8	-	-	-	-
Treatment plans and interventions	8	-	-	-	-
Next visit date	6	1	1	-	-
**Examination **					
Physical examination	8	-	-	-	-
Neurological evaluation	7	1	-	-	-

Abbreviations: CT, computed tomography; MRI, magnetic resonance imaging; EMG, electromyography; BMD, bone mineral density; CBC, complete blood count; CRP, C-reactive protein; ESR, erythrocyte sedimentation rate; LDL, low-density lipoprotein; TSH, thyroid stimulating hormone.

**Figure 2.**
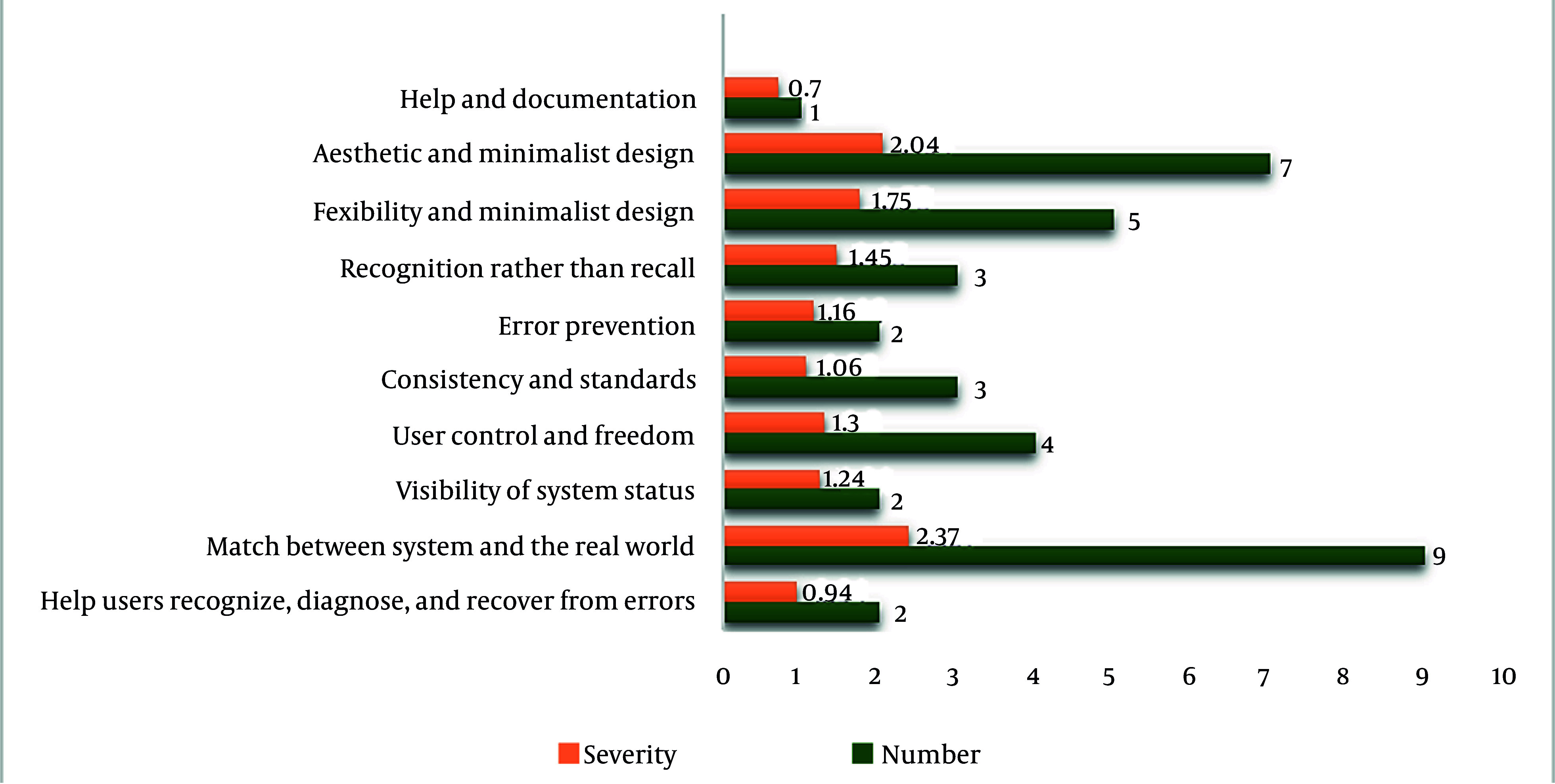
The number and severity of problems identified by the Nielsen method

In the second phase, the initial version of the system was designed and provided to three evaluators. The highest number of problems (n = 9) and the highest mean severity score (2.37 on a 0 - 4 scale) were observed in the heuristic category "Match Between System and the Real World". Figure 2 shows the number and severity of problems identified by the Nielsen method.

After resolving the identified issues, the final version of the system was implemented and made available to users. Users must register and verify their identity as a patient, specialist physician, or system administrator to use the system. In the next step, the user can log in to their account by entering their username and password.

The patient panel includes a dashboard, electronic medical record, profile management, and support. The patient accesses the request registration section through the electronic medical record. Initially, they enter the system's demographic information, medical history, and pain history. If they have any previous paraclinical tests or laboratory results, they upload those as well. Subsequently, they select the type of consultation and their preferred physician based on the available appointment slots. Finally, by completing the consent form and making the payment, the patient's request is registered, a confirmation SMS is sent to them, and their file is referred to the specialist panel.

**Figure 3.**
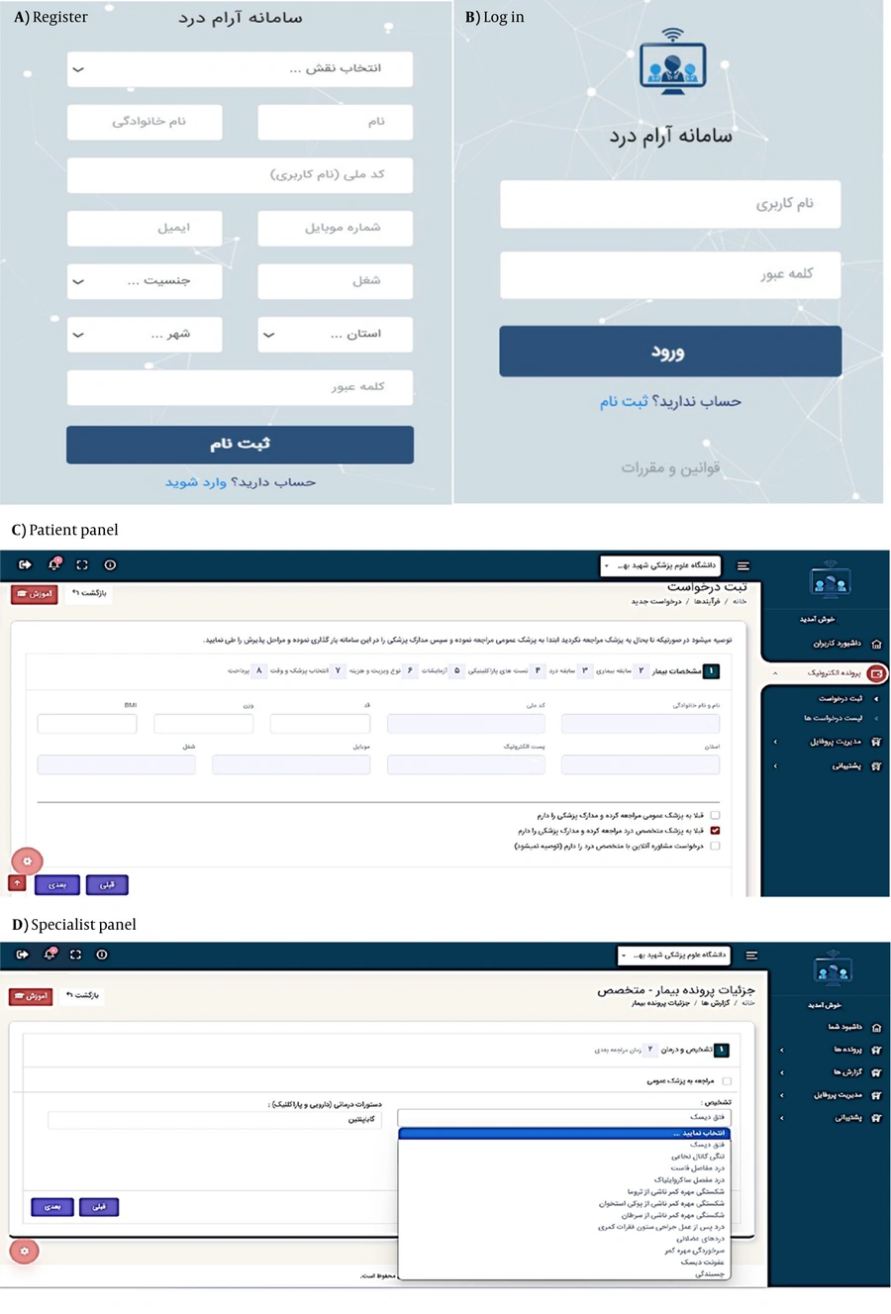
The various components of the telemedicine system

The specialist physician panel includes a dashboard, patient records, reports, profile management, and support. The specialist physician selects the patient from the list of records and clicks on the "View Record" option to access the demographic information, medical history, clinical data, and results of laboratory and paraclinical tests, and enters the diagnosis and treatment page by selecting the "Action" option. The specialist physician may refer the patient to a general practitioner for further examinations. In this case, the patient must log into their user panel and view the list of requests to download the general practitioner form, which includes physical examinations, neurological assessments, preliminary diagnoses, paraclinical tests, and laboratory studies. The patient should then visit the general practitioner to complete the form. Subsequently, they need to enter the form information into the system and send it to the specialist physician. Alternatively, the specialist physician may make the diagnosis and treatment decisions based on the patient's record. If the patient requires follow-up and monitoring, the specialist physician navigates to the follow-up section and specifies the date and time for the next appointment. The specialist physician may refer the patient to an orthopedic specialist, psychiatrist, or other healthcare provider. The different components of the telemedicine system are illustrated in Figure 3. 

The results of the usability evaluation of the telemedicine system are shown in Table 3. The highest score for both specialist physicians and patients was obtained in "Learning". The total scores for specialist physicians and patients were 8.07 ± 0.41 and 7.73 ± 0.55, respectively. Overall, the level of satisfaction with the system's user interface was good.

**Table 3.** Results of Telemedicine System Usability Evaluation ^a^

Measure	Participants	
	Patients	Specialist Physicians
**Screen**	7.63 ± 0.34	7.45 ± 0.59
**Terminology and system information**	7.56 ± 0.91	8.16 ± 0.32
**Learning**	8.34 ± 010	8.75 ± 0.06
**System capabilities**	7.09 ± 0.42	7.55 ± 0.83
**Overall reactions to the software**	8.04 ± 0.75	8.45 ± 0.26
**Total**	7.73 ± 0.55	8.07 ± 0.41

^a^ Values are expressed as mean ± SD.

## 5. Discussion

This study aimed to design a telemedicine system for chronic pain management, which was conducted in three phases. Considering the importance of determining the minimum data set in the present study, essential data elements for a telemedicine system for chronic pain management were extracted through a literature review and the experiences of other countries in system development. Our study results indicated that the minimum data set for designing a telemedicine system for chronic pain management comprises 56 elements across 8 main categories. These categories include demographic information, patient history, pain history, laboratory tests, paraclinical tests, psychological assessments and daily living activities, diagnostic and therapeutic actions, and examinations.

The National Institutes of Health (NIH) provided the minimum data set in four categories: Demographic information, medical history and examinations, pain description, and mental health and daily living activities (32). In the study by Rosenquist et al., the minimum data set comprised 37 elements across four domains: Demographic and social data elements, medical history and examination data elements, pain data elements, and psychological assessments and daily living activities data elements (33). In the study by Baradaran et al., the initial minimum data set consisted of 51 elements, which were reduced to 41 elements after expert review. This set included six domains: Demographic information, initial pain assessment, medical history, mental health and well-being, diagnostic actions, and the diagnostic and treatment plan (29). The findings of these studies align with the present study. However, since the present study aims to design a system and create a panel for patients and specialists, there are differences in the categorization of topics compared to other studies.

During the evaluation phase of the system's adherence to usability principles, three evaluators assessed the features of the telemedicine system using Nielsen's ten standard and predefined principles. After consolidating and summarizing the problems identified by the three evaluators and removing duplicate items, 38 unique problems remained. Most of the problems were related to violating the principle of "Match Between System and the Real World", primarily due to the novelty of telemedicine in Iran and the limited exposure of both patients and some specialists to digital health platforms. Such unfamiliarity likely influenced the expectations and interactions of the users with the system, leading to difficulties when the system's working routines did not perfectly fit actual practice. These findings highlight the importance of user-centered design, iterative testing, and targeted training programs to enhance usability and acceptance. For the broader adoption of telemedicine in Iran, it is crucial to provide sufficient guidance and support to both patients and healthcare providers, ensuring that digital systems reflect real-world clinical contexts as closely as possible.

In the study by Lilholt et al., five evaluators assessed the Telekit system and identified 86 problems. The most prevalent problem was "Consistency and Standards" (34). In the study by Aldekhyyel et al., three telemedicine applications (Seha, Cura, and Dr. Sulaiman Alhabib) were evaluated, and a total of 54 problems were identified. For the Seha application, the most significant problems were related to "User control and freedom" and "Recognition rather than recall". In the Cura application, the most significant problems were related to "Consistency and standards", "Aesthetic and minimalist design", and "Help and documentation". In the Dr. Sulaiman Alhabib application, the most significant problem was related to "Error prevention". According to the findings of the present study, "Aesthetic and minimalist design" had the most problems, which aligns with the findings from the Cura study (35). However, it is important to note that these variations in research findings are interpretable due to the differences in the studies, their objectives, and the system capabilities.

In the present study, during the evaluation phase of the user interface, the highest score for both specialists and patients was achieved in the item "Learning". This could be attributed to the fact that users participated in a one-hour training session before starting to use the system. Additionally, they were provided with an instructional PDF file detailing how to operate the system. Furthermore, to facilitate the use of the system, a mobile phone number was made available for addressing any potential problems during its use. The lowest score for specialists was related to the item "Display Screen", while for patients, it was related to "System Capabilities". These findings indicate a good level of acceptance and usability of the telemedicine system's user interface.

User satisfaction with the telemedicine system's user interface has also been examined in numerous studies. According to Mohanraj et al., the highest score was attributed to "System Capabilities" in their study. Scores for "Learning", "Overall Software Responsiveness", "Display Screen", and "System Terminology and Information" were ranked next in subsequent categories. Overall, the results indicated that users rated the system's usability at a good level (36). The findings of this study are consistent with the present research in that satisfaction levels are assessed to be good.

### 5.1. Conclusions

As chronic pain becomes more prevalent among underserved and isolated populations, it is increasingly important to develop adaptable care models that can support large numbers of patients. Tailored telemedicine can be used to connect patients with medical care when access is limited and help bring together different medical experts to collaborate on pain care. This study contributes to the field by demonstrating that establishing a tailored telemedicine platform for managing chronic pain in Iran is feasible. Using a system based on needs assessment by patients and medical staff, designed with user input and implemented in real practice, the platform provided remote consultations, health data access, and opportunities for patient participation. The observation that most providers and patients are satisfied with the system indicates that it has been both acceptable and usable for all users.

However, digital health solutions are not successful if they rely solely on the technology's implementation. For healthcare IT to be successful in the long run, it should be integrated with national electronic health records, involve training stakeholders, support digital literacy, and be covered by laws protecting patient data. Assessing the impact of the procedure on pain levels, patient functionality, treatment compliance, and interactions with clinicians should be validated through large, rigorous studies.

Overall, while this study makes a promising start toward managing chronic pain with technology and a patient-focused approach, it also highlights the numerous challenges involved in transforming how care is delivered. Enhancing inclusive design, robust infrastructure, and evidence-based assessment is necessary for making telemedicine effective in pain care and other areas.

### 5.2. Limitations

The determination of user needs was mainly limited to Shahid Beheshti University of Medical Sciences. Efforts were made to extend the scope of the view by involving other institutions in seeking expert views via telephone interviews and social media, but the generalizability of these findings is inherently limited. This, in turn, means that the applicability of these findings is limited in itself. Further studies at various pain clinics in different healthcare facilities would be useful to confirm and build on these findings, which may bring in different findings.

## Data Availability

The datasets used and/or analyzed during the current study are available in the article.
